# Perceptions of cervical cancer care among Ethiopian women and their providers: a qualitative study

**DOI:** 10.1186/s12978-021-01316-3

**Published:** 2022-01-04

**Authors:** Sahai Burrowes, Sarah Jane Holcombe, Cheru Tesema Leshargie, Alexandra Hernandez, Anthony Ho, Molly Galivan, Fatuma Youb, Eiman Mahmoud

**Affiliations:** 1 Touro University California, Vallejo, CA USA; 2 Bill & Melinda Gates Institute for Population and Reproductive Health, Johns Hopkins Bloomberg School of Public Health, Baltimore, USA; 3 Debre Markos University, Debre Markos, Ethiopia

**Keywords:** Cervical cancer, Ethiopia, Quality of care, Patient experience, Stigma

## Abstract

**Background:**

Cervical cancer is the second most commonly diagnosed cancer among Ethiopian women, killing an estimated 4700 women each year. As the government rolls out the country’s first national cancer control strategy, information on patient and provider experiences in receiving and providing cervical cancer screening, diagnosis, and treatment is critical.

**Methods:**

This qualitative study aimed to assess the availability of cervical cancer care; explore care barriers and sources of delay; and describe women’s and providers’ perceptions and experiences of care. We analyzed data from 45 informants collected at 16 health centers, district hospitals and referral hospitals in East Gojjam Zone and a support center in Addis Ababa. Thirty providers and ten women receiving care were interviewed, and five women in treatment or post-treatment participated in a focus group discussion. Deductive and inductive codes were used to thematically analyze data.

**Results:**

Providers lacked equipment and space to screen and treat patients and only 16% had received in-service cervical cancer training. Consequently, few facilities provided screening or preventative treatment. Patients reported low perceptions of risk, high stigma, a lack of knowledge about cervical cancer, and delayed care initiation. All but one patient sought care only when she became symptomatic, and, pre-diagnosis, only half of the patients knew about cervical cancer. Even among those aware of cervical cancer, many assumed they were not at risk because they were not sexually active. Misdiagnosis was another common source of delay experienced by half of the patients. Once diagnosed, women faced multiple-month waits for referrals, and, once in treatment, broken equipment and shortages of hospital beds resulted in additional delays. Barriers to therapeutic treatment included a lack of housing and travel funds. Patient-provider communication of cancer diagnosis was often lacking.

**Conclusions:**

In-service provider training should be intensified and should include discussions of cervical cancer symptoms. Better distribution of screening and diagnostic supplies to lower-level facilities and better maintenance of treatment equipment at tertiary facilities are also a priority. Expanded cervical cancer health education should focus on stigma reduction and emphasize a broad, wide-spread risk of cervical cancer.

## Plain Language Summary

Cervical cancer is the second most commonly diagnosed cancer among Ethiopian women, killing an estimated 4700 women each year. This study aimed to assess patient and provider experiences in receiving and providing cervical cancer screening, diagnosis, and treatment. We interviewed 30 midlevel providers and ten women receiving care and held a focus group discussion with five women who were receiving treatment or who had recently completed treatment. Patients reported bottlenecks and delays at each stage of care. Low perception of risk, high stigma, and a lack of knowledge about cervical cancer among both providers and patients, were significant sources of delay in initiating care. Few patients had been aware of cervical cancer before they were diagnosed and of those who were aware, many assumed that they were not at risk because they were not sexually active. Misdiagnosis was another common source of delay. Once diagnosed correctly, women faced multiple-month delays after referrals, and, once in treatment, broken equipment and a shortage of hospital beds resulted in additional delays. The most frequently mentioned barriers to care were a lack of housing and travel funds while receiving treatment in the capital. Patient-provider communication of cancer diagnosis was often poor. Our findings suggest the need to intensify in-service training for providers, focusing initially on alerting them to cervical cancer symptoms. Better distribution of screening and diagnostic supplies to lower-level facilities and better maintenance of treatment equipment at tertiary facilities should also be a priority.

## Background

Cervical cancer is the second most common cancer in women worldwide and in Ethiopia [1, 2]. Human papilloma virus (HPV) vaccination and routine and well-resourced screening programs linked to preventive and therapeutic treatment services can markedly reduce cervical cancer-related deaths and illness [3, 4], as has occurred in many high-income countries, where such services are estimated to prevent up to 80% of cervical cancers [5]. However, in low-income countries such as Ethiopia, low levels of public awareness, limited or non-existent HPV immunization and screening programs, and insufficient health infrastructure contribute to most women either never being immunized or screened or being screened late. Accordingly, between 60 and 80% of cervical cancer cases are diagnosed in advanced stages (i.e., stages 3 and 4) [1, 6–9]. Because of these limitations, the global incidence of cervical cancer continues to rise, with the overwhelming preponderance of the burden in low- and middle-income countries, where over 90% of deaths from cervical cancer occur [7, 10, 11].

**Fig. 1:**
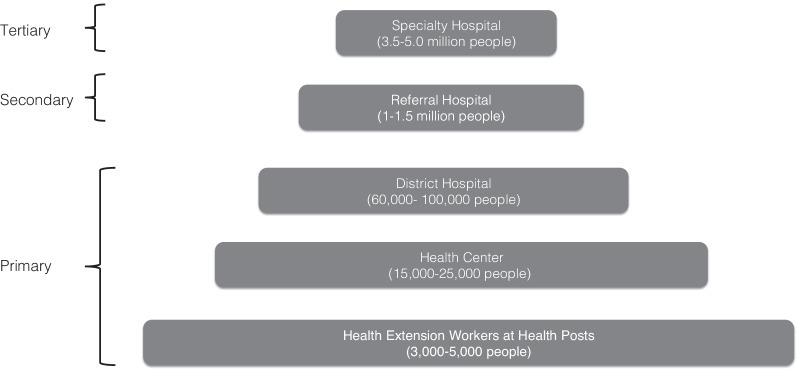
Ethiopia’s Three-tiered Health System. Adapted from the Government of Ethiopia’s Health Sector Transformation Plan [15]

In Ethiopia, it is estimated that over 6000 new cervical cancer cases and over 4700 cervical cancer deaths occur annually [12]. These figures are acknowledged as underestimates due to the absence of systematic immunization or screening programs or a national cancer registry [13]. In response to this health threat, Ethiopia’s 2015 National Cancer Control Plan aimed to control cervical cancer, in part through the introduction of a national cervical cancer screening program using a “screen and treat” approach—visual inspection of the cervix with acetic acid (VIA), followed by immediate cryotherapy preventive treatment for any women with precancerous lesions detected. Under the plan, screen and treat services are offered at primary care facilities (see Fig. 1). Women with more advanced cervical cancer (stages 3 and 4) are to be referred to secondary and tertiary facilities for further diagnostic and therapeutic treatment services [14]. The plan also institutes measures to strengthen diagnosis, referral, and treatment.

Ethiopia’s screening program is still relatively new, and treatment services are limited. As in most sub-Saharan African countries, Ethiopia’s efforts are hampered by the lack of trained workers, equipment, or effective referral systems at primary care health centers and district-level hospitals—infrastructure essential for screening women and treating those diagnosed with invasive cancer [5, 16]. Even in the capital, Addis Ababa, health facilities almost overwhelmingly lack basic cervical cancer treatment equipment [17]. Tikkur Ambessa (Black Lion) Hospital is the country’s main public cancer referral facility and the only one with radiotherapy and is severely over-burdened [18, 19]. In the 2008–2012 period, almost 40% of the 2300 patients with cervical cancer who registered at the radiological clinic at Tikkur Ambessa never received therapy [20].

The National Cancer Control Plan aims to control cervical cancer by, in part, scaling up screening services and reducing gaps between screening and treatment [13]. To do so, policymakers require information about the availability, strengths, and weaknesses of diagnosis and treatment services, as well as about how women navigate these services. The Lancet Global Health Commission has called for more research in low- and middle-income countries on care quality for cancer and other understudied conditions, and has described user experience as a particular blind spot [21]. However, in Ethiopia, little is known about women’s actual experience accessing cervical cancer screening, diagnosis, and particularly treatment services, or about how women transition from one service to another.

Research on cervical cancer interventions in Ethiopia has focused almost exclusively on women’s awareness and knowledge of cervical cancer and screening [8, 16, 18, 22, 23], and to a lesser extent on provider perspectives on screening [17, 23, 24]. The knowledge about causes, prevention, and treatment of cervical cancer among women of reproductive age has typically been found to be limited [9, 22, 25, 26], and the prevalence of screening exceedingly low [16, 27], although at least one study encountered high levels of support among rural women for cervical cancer screening [26].

Among Ethiopian health care workers, studies have found knowledge of cervical cancer to be high [24, 28], and a strong belief that cervical cancer screening is an essential part of women’s health care [24]. However, this knowledge may still be patchy and unevenly distributed among health care providers, with nurses, midwives, and health officers having significantly lower cervical cancer knowledge than physicians [29]. Most health care workers lack training in the provision of cervical cancer care, and fewer still have provided screening [24, 28, 30]. Perhaps unsurprisingly, one study showed that few (< 12%) female Ethiopian health professionals themselves have been screened [28].

In contrast with the growing literature on knowledge, attitudes, and practices relating to cervical cancer in general, and screening in particular, very little research has been done on women’s *experience* of cervical cancer screening or of treatment and care post-screening. A systematic review finds that, as of 2016, fewer than 14 studies had been conducted on women’s treatment experience in sub-Saharan Africa [5] and only one in Ethiopia, which focused specifically on palliative care [31]. Of the five more recent articles we found that touched on the treatment of cervical cancer in Ethiopia, only one examined patients’ care experience [23], and it did not cover delays in treatment, referrals, or provider communications.

In light of this gap in the literature, this study sought to better understand experiences of cervical cancer screening, diagnosis, and treatment in Ethiopia. We had three research aims. The first was to describe the availability of cervical cancer screening, diagnostic, and treatment services in the study area and to assess the strength of referral systems between these services. The second was to document barriers to care and sources of delay that women faced before and after screening. And the third was to describe women’s perceptions and experiences of cervical cancer care.

## Methods

### Study design

**Fig. 2:**
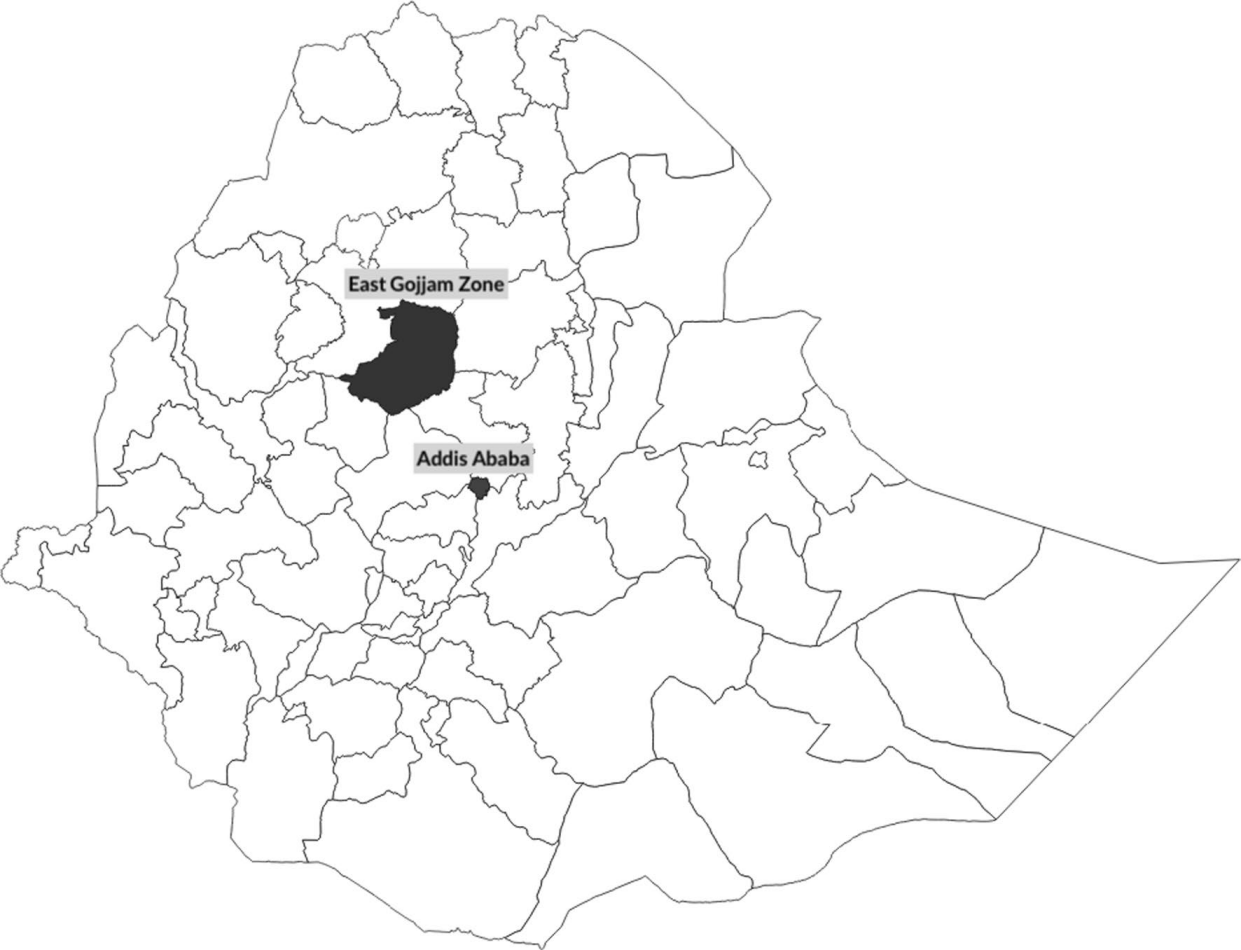
Map of Ethiopia with Project Areas Highlighted

This is a qualitative study using interview and focus group data from medical providers and patients in rural and urban areas of the East Gojjam Zone in northwestern Ethiopia and from Addis Ababa (see Fig. 2). To address our first research aim on service availability, we conducted semi-structured in-depth interviews with health care providers (n = 30). To address our second and third research aim we conducted seven semi-structured in-depth interviews with women presenting at health facilities with reproductive health complaints in Debre Markos and three interviews and a focus group discussion with women who were either undergoing treatment for cervical cancer or who had recently completed treatment in Addis Ababa (n = 8). For our second research aim, we triangulated women’s data on perceptions of barriers to care and sources of delay with data from providers.

### Sampling strategy

#### Health care providers

The provider interviewee sample was drawn from clinical staff at 16 of the 120 primary, secondary, and tertiary referral facilities located in East Gojjam Zone, Amhara Regional State, a primarily rural area of northwest Ethiopia (see Fig. 2). Study facilities included a purposive sample of the two referral hospitals in the zone (Debre Markos Referral Hospital [DMRH] and the Felege Hiwot Referral Hospital); a purposive sample of all four urban primary care health centers in the town of Debre Markos; and ten randomly selected primary health centers and primary district hospitals in the catchment areas of the referral hospitals (see Table 1). Randomization was conducted using a random number generator in the software program Stata to select facilities from a numbered list of primary health facilities in the zone.

**Table 1:** Type of health facility sampled

Facility type	Number
Referral Hospital	2
Primary (District) Hospital	4
Rural Health Center	6
Urban Health Center (Debre Markos Town)	4
Total	16

We recruited a convenience sample of two to three healthcare providers at each facility from the clinical staff that was on duty. All providers who had cervical cancer screening and care in their scope of practice as laid out in the Ministry of Health’s guidelines, who were over the age of 21, who were on duty at the time of recruitment, and who were currently providing reproductive health services at a study facility, were eligible for inclusion. This included midwives, nurses, health officers, pathologists, and obstetrician-gynaecologists.

#### Women receiving care

Women receiving care were recruited from two geographic areas. A convenience sample of seven women at the early stages of their cervical cancer care experience was recruited from the sampled East Gojjam Zone health facilities (see Table 3). In order to include women at advanced stages of their treatment experience, we also recruited three additional interviewees and five focus group participants from the capital, Addis Ababa, where treatment services are located. These women were either undergoing treatment or had recently completed treatment at Tikkur Ambessa Hospital and were receiving supportive care services at the Mathiwos Wondu-YeEthiopia Cancer Society (MWECS) support center.

The inclusion criteria for these interviewees and focus group members were that they be females over age 21 who had either presented at a study health facility for cervical cancer screening, or with a reproductive health complaint that could be suspected cervical cancer or pre-cancer or who were either currently receiving cervical cancer treatment at Tikkur Ambessa Hospital or had recently completed treatment there (see Table 2). We limited the participation of women with HIV-infection to less than half of the sample of women receiving care, as such women experience more rapid and acute disease progression [32], and may be eligible to access additional health education and care services [18]. We did not track the number of women or providers who refused to participate in the study.

**Table 2:** Sampling strategy by participant type and methodology

Participant type	Research aim	Data collection methodology	Study site	Sampling strategy
Health care providers (nurses, midwives, health officers physicians) (n = 30)	1 & 2	Short structured interview	Health facilities in East Gojjam Zone	Convenience sample in purposively and randomly selected facilities
Women receiving care (presenting for screening or with symptoms) (n = 7)	2 & 3	In-depth interview	Health facilities in East Gojjam Zone	Convenience sample
Women receiving care (undergoing or having recently completed treatment) (n = 8)	2 & 3	In-depth interviews (n = 3) Focus group discussion (n = 5)	MWECS^a^ support center in Addis Ababa	Convenience sample

^a^MWECS is the Mathiwos Wondu-YeEthiopia Cancer Society

East Gojjam Zone patients were recruited through the distribution of study invitation flyers at the 16 facilities where providers were interviewed, as well as through onsite recruitment by project recruiters at eight of the sampled facilities. A MWECS manager recruited interviewees and focus group participants in Addis Ababa using center announcements and phone calls to patients on the center’s registries.

### Data collection instruments

The pre-tested interview guide for health care providers included questions on training, level of comfort, and experience in carrying out cervical cancer screening, diagnosis, and treatment services, as well as systems for referrals and follow-up visits, supportive services available to women, and recommendations for improving care.

Patient in-depth interviews and the patient focus group used structured and pre-tested interview and focus group guides with questions on women’s history of diagnosis and treatment; their access to supportive services; the barriers they faced; and their perceptions of the quality of care they had received, including the quality of interactions with providers.

All data collection was conducted in Amharic or Afaan Oromoo. Patient data collection instruments and informed consent materials were translated into Amharic and Afaan Oromoo by a professional translator and crosschecked by a second translator.

### Data collection

Data were collected from March 2017 to July 2017. East Gojjam Zone interviewers (one cis-gender female, one cis-gender male) and recruiters (two cis-gender males) were recent graduates of the Debre Markos University School of Public Health program. They received a 4-day training at Debre Markos University conducted by local experts, covering interviewing skills, interaction with patients with serious illness, data management, and research ethics. The additional individual interviews and the focus group with women receiving supportive care in Addis Ababa were conducted at MWECS by two professional qualitative researchers from Addis Ababa University (both female), in Afaan Oromoo and Amharic, respectively.

All provider and patient interviewees and focus group participants gave oral informed consent to participate and to be recorded just prior to data collection. At the time, the aims of the study were also explained, but no other information regarding biases or personal interest in the study was shared. The data collectors had no relationship with study participants prior to the start of the study.

The provider interviews took place in private rooms at the sampled East Gojjam Zone health facilities. In-depth interviews with the East Gojjam Zone patients also took place at study health facilities or outside away from the main building, after study recruiters had explained the study and the interviewers had obtained informed consent. Researchers attempted a follow-up phone interview with these women six weeks after the initial interview, but most women were not available for these calls due to illness or poor phone connectivity so this portion of the study was abandoned. The focus group discussion and the three Addis Ababa-based interviews were conducted in a private room at MWECS. Only a single interviewer was present for each interview. A focus group facilitator (young, cis-gender, female) and her notetaker (older, cis-gender female) were the only researchers present during the focus group discussion.

All interviews and focus group discussions were audio-recorded with permission from participants. Interviews were approximately 30 min on average, and the focus group discussion was approximately an hour and a half. Field notes were not taken during the interviews and focus groups, but after each interview or focus group, a debrief summary form was completed to note the context of the encounter and any outstanding points as well as to summarize the respondents’ statements.

Audio files were simultaneously transcribed and translated into English by a professional translator. Transcripts were not returned to participants for comment or correction, and participants did not give feedback on findings. All analyses were conducted in Dedoose. A team of three Masters in Public Health student research assistants (one cis-gender male, two cis-gender females) and the project investigators (both cis-gender females with PhDs in Health Policy) coded transcripts using both a priori codes based on our research aims, as well as inductive codes emerging from initial readings. The teams defined saturation as likely been reached when no new themes emerged from the last transcript. Codes were collapsed into broad themes and respondent quotes selected to illustrate these themes (i.e., thematic content analysis). We selected quotes that were the most eloquent and the most representative of the data; therefore, not all respondents are quoted in the results to follow and some, particularly expressive, respondents may be quoted more than once.

## Results

### Sample characteristics

#### Health care providers

Providers in our sample had an average age of 29 years and an average of 7 years of professional experience (see Table 3). The majority was male.

**Table 3:** Characteristics of health care providers (n = 30)

Characteristics	n	%^c^
Age category		
30 and under	24	80
31–40	4	13
40+	2	7
Gender		
Female	9	30
Male	21	70
Father’s educational level^a^		
No formal schooling	14	47
Some primary	9	30
Some secondary school	3	10
Completed secondary school	3	10
Information missing	1	3
Profession		
Health officer^b^	9	30
Midwife	12	40
Nurse	6	20
Physician	3	10
Facility type		
Health Center	18	60%
District Hospital	7	23%
Referral Hospital	5	17%
Years worked in the profession		
Fewer than 5 years	11	37%
5–10 years	15	50%
More than 10	4	13%
Years at current position		
Less than 1 year	5	17%
1–2 years	8	27%
3–5 years	12	40%
More than 5 years	5	17%

^a^Father’s educational level is a proxy for the respondent’s family socioeconomic status

^b^Health Officers are advanced practice clinicians, similar to physician assistants in the United States, who provide clinical services, including basic obstetric surgeries primarily at health centers and district hospitals, and who often manage families and district-level ministry of health offices

^c^Percentages may sum to more than 100% due to rounding

#### Women receiving care

Most women receiving care in the sample were either in treatment for cervical cancer or had completed treatment (see Table 4). Women’s average age was 40. Approximately one-third was HIV-positive. Most were married, lived in urban areas or small towns, and were from modest socioeconomic backgrounds.

**Table 4:** Characteristics of women receiving care (n = 15)

Characteristic	n	%
Age category		
Age 30 & under	3	20
Age 31–40	4	27
Older than 40	8	53
Martial status		
Divorced	3	20
Married	6	40
Single	1	7
Information missing	5	33
Residence		
City	4	27
Small town	5	33
Rural	3	20
Information Missing	3	20
Father’s education^a^		
No Schooling	6	40
Primary School	5	33
Secondary School	2	13
Seminary training	2	13
Ethnic group		
Amhara	8	53
Oromo	5	33
Wolaitta	2	13
HIV-status (self-reported)		
HIV-positive	6	40
HIV-negative	9	60
Stage of care		
Initial screening: asymptomatic	1	7
Initial screening: symptomatic	1	7
Post diagnosis, advanced cancer: awaiting treatment	5	33
Advanced cancer: in treatment	1	7
Advanced cancer: post-treatment	7	47

^a^Father’s educational level is used as a proxy for the respondent’s family socioeconomic status

### Overview of themes

The main themes and sub-themes found in the data reflected both our research aims (e.g., barriers to care, sources of delay, low service availability) and emergent trends (e.g., poor provider-patient communication). Themes are summarized in Table 5 and discussed below.

**Table 5:** Themes from interviews and focus groups

Theme	Sub-themes & triangulation	
	Providers	Women receiving care
Limited availability of services at the primary care level	Lack of in-service training on cervical cancer and screening Lack of infrastructure and supplies for screening and treatment *Lack of supportive services for cervical cancer patients*	Women leapfrog primary care facilities to seek care from secondary facilities *Lack of supportive services for cervical cancer patients:* family as main support
Weak referral networks	Few systems in place to follow up on referred patients Ad hoc systems used to track patients	
Barriers to initiating and continuing care	*Patients’ low awareness and low perception of risk* *Lack of finances and transport for follow up care/referrals*	*Patients’ low awareness and low perception of risk:* Women do not think they are at risk if they are monogamous or not sexually active *Lack of finances and transport for follow up care/referrals* Stigma, which delays care initiation and seeking follow-up care Societal barriers, namely rural women’s need for partner approval to seek care
Sources of delay in accessing appropriate treatment	Loss to follow-up after referrals, mainly due to financial barriers and *long wait times for referral appointments*	Misdiagnosis of cervical cancer Lack of equipment, laboratory services, and beds *Long wait times for referral appointments*
Patient-provider communication	Difficulty communicating due to shy and uncomfortable patients	Unclear or missing communication, particularly about diagnosis Power imbalance and fear of repercussions in asking questions Difficulty communicating due to language barriers
Recommendations for change	*Expand and decentralize the provision of services:* Increase training, availability of supplies, and equipment at local level *Expand health education and outreach* for the general public and leaders	*Expand and decentralize the provision of services:* Increase service availability at local level *Expand health education and outreach,* especially for rural community members

Italicized sub-themes were found in both patient and provider data

### Theme: limited service availability

#### Sub-theme: no screening or preventative treatment provided

Providers reported that there were few screening, diagnostic, or treatment services offered in East Gojjam Zone outside of secondary referral facilities. When asked about the screening services that they offered, most providers at primary facilities reported that they provided symptom assessment (sometimes accompanied by unassisted visual inspection) and referrals for further screening or diagnosis.

*We don’t treat here, and if we suspect the case to be cervical cancer based on the symptoms, we refer to better facilities* (Midwife 1, Health Center).

*It is difficult to even say [that we do] screening. We review clinical symptoms only and refer them. We don’t have the instruments to screen* (Midwife 2, District Hospital)

Providers reported offering VIA at only four of the 14 primary care-level facilities, and only one primary care facility was reported as offering cryotherapy. None offered diagnostic or therapeutic treatment services. The two referral hospitals in our sample did offer a comprehensive suite of preventive and diagnostic services and preventive treatment (i.e., cryotherapy).

#### Sub-theme: patients leapfrog primary care

Although patients did not mention the lack of services at lower-level facilities directly, most mentioned initiating care either at a secondary referral hospital or in the private sector, indicating that they did not believe that public primary facilities could help them. In addition, their recommendations for improving care centered on increasing services at the community level.

*Because if services were available at hospitals close to us, we would not have to incur transportation and other unnecessary costs in addition to all the hardships we face to get to the place of referral. If that was the situation, you see, we could get care close to our homes and be close to our families. In addition, I think it would be positive if more hospitals and health facilities were built and staffed to avoid long waits* (Patient 1, Currently in treatment, Interview)*.*

#### Sub-theme: lack of infrastructure

When explaining the lack of cervical cancer services at their facilities, providers stated that most facilities lacked the equipment, supplies, or private space necessary for screening or diagnosis.

*The first [gap] is a lack of a trained workforce. Even the trained ones, like me, are not providing services. In Debre Markos Hospital, they have a separate examination room, instruments are available, and the service is being provided on the spot. Here there are space and supply-related challenges* (Nurse 1, Rural Health Center).

#### Sub-theme: lack of training

Lack of training was another common reason cited for the low level of service provision. Only half of providers interviewed had received in-service training on cervical cancer screening and preventative treatment, and two-thirds said that they had low confidence in their ability to provide these services. Most midlevel providers had no in-service training on cervical cancer. Several providers reported that they learned about cervical cancer on their own by reviewing textbooks and pamphlets.

*I have some knowledge based on the clients’ problems that I encounter, but I didn’t have any training* (Health Officer 3, Rural Health Center)

*I have downloaded some reference documents from the internet. I also use Harrison’s text book [of internal Medicine] shared by my friend. We also download gynecological text books* (Health Officer 2, Urban Health Center)

#### Sub-theme: lack of social support services

In addition to the lack of clinical services for women with suspected or invasive cervical cancer, social support services such as transportation assistance, financial support, or palliative or home care were largely absent from facilities. Only three facilities sampled linked patients to transport, financial or housing funds, and these often seemed to be ad hoc initiatives of individual health care providers rather than systematic programs.

*Sometimes, when they can’t get transportation, we facilitate taking them at least to Motta [Town], on their way to Bahir Dar Referral Hospital.* (Health Officer 1, District Hospital)

When describing their course of treatment, several women receiving care appreciatively highlighted health providers who went out of their way to provide ad hoc transport, counseling, or other types of support in an attempt to connect them to further treatment.

*The health care provider …took me to Black Lion Hospital with his own car and covered the different financial issues that I had. I would like to thank him* (Patient 13, Treatment completed, Interview).

However, women’s predominant sources of social support were family, friends, and the MWECS support center.

*My children help me a lot and support me with all they have and they took care of me every time I was sick and couldn’t take care of myself. And also my neighbors, they support me like they are my own family* (Patient 4, Awaiting treatment, Focus Group).

### Theme: weak referral and follow-up systems

#### Sub-theme: lack of formal feedback and follow-up

Most providers commented on the shortcomings of referral arrangements. Further, they noted that these systems were slow and lacked mechanisms for follow-up and feedback. A third of providers commented on feedback mechanisms, reporting that once they had made referrals, they had no way of knowing whether their patients were able to access follow-up care, let alone what types of care they were receiving. Providers who had referred patients for follow-up care were often exasperated on their behalf.

*To begin with, no one sends referral feedback to you from Addis Ababa and Bahir Dar. Forget giving feedback, they do not even answer your phone call! I called a provider two times and on the third call, after he knew my number, he didn’t answer…. Even though I don’t get referral feedback, as the number of my clients is small, I call to them and ask about their status*. (Midwife 3, Referral Hospital)

*To tell the truth, we didn’t have it as a routine practice to track status of all referred cases.* (Health Officer 4, Urban Health Center)

#### Sub-theme: Ad hoc follow up systems

Informal phone communication was mentioned by several providers particularly those at larger facilities, and, appreciatively, by patients, as a way of following up on referrals.

*We exchange telephone numbers with our patients and advise them to communicate with us when they reach there. We also contact the cervical cancer service provider to receive the referred cases by indicating the name of the client and date of the referral* (Physician 2, Referral Hospital)

### Theme: barriers to care

#### Sub-theme: low risk perception and lack of knowledge

Providers identified women’s low risk perception and limited awareness of cervical cancer as one of the chief barriers to seeking and entering care.

*Due to low levels of community awareness, most people do not come for routine screening and most patients come once the disease has advanced.* (Physician 3, Referral Hospital)

The providers’ sentiments were corroborated by the finding that only half the women participating in the study had ever heard of cervical cancer pre-diagnosis. Almost all women initiated care because they were symptomatic. The non-symptomatic women who initiated screening had all received direct health education, either through the community or, more often, from their HIV treatment counselors. Those who had heard of cervical cancer prior to their diagnosis assumed that they were not at risk because they were not sexually active or were monogamous.

*I had heard about [cervical cancer] when people from the government came to Hawassa once to call up on us to get checked... But I did not think I would get it as I was not having any sexual relations …* (Patient 14, Treatment completed, Focus Group).

*I did not think I would have such a disease, you see this girl died from it and I just assumed that she had the disease because she went out with many men. How could I imagine that one could get it just living their lives?* (Patient 7, Awaiting treatment, Focus Group).

Notably, although low awareness and low risk perception emerged from patients’ stories as barriers to care no patients *directly* mentioned their lack of awareness or low risk perceptions about cervical cancer as barriers for entering care.

#### Sub-theme: stigma

Patients described stigma as a substantial barrier to care. Patients said that they delayed seeking care because they felt both ashamed and hopeless about their potential diagnosis. They perceived cervical cancer to be viewed extremely negatively by their communities, particularly as it is thought to be caused by promiscuity or to be a punishment from God. Relatedly, some women also indicated that they had been reluctant to initiate treatment due to shame surrounding their condition.

*My main barrier [to care] was community perceptions. Until I came here [MWECS support center] and saw other women who had similar problems like me, I had a severe psychological problem after I found out that my problem was cervical cancer; because in my community I never heard or saw such cases before. I was worried that the community would discriminate against me and stigmatize me when they heard about my problem. I felt sadness until I came here and knew there were other women who had similar problems to me.* (Patient 11, Awaiting treatment, Interview)

*Well, what I want to say a little bit about is the attitude of the community towards cervical cancer. People see cervical cancer as something so different from other illnesses and something that is so terrible, I mean to say that the society has the belief that a person who has this illness is cursed from above and that this person is a sinner who has done something to upset God* (Patient 1, Currently in treatment, Interview)

#### Sub-theme: societal barriers

Some also cited as a barrier women’s need to gain permission from husbands for obtaining care, and the views of some husbands that no care is warranted unless women are incapacitated.

*For example, there are families of farmers and the husbands will not allow his wife to leave her work and go anywhere. So long as she is able to walk he will not be convinced that she is sick. For him to believe that she is sick she must be unable to walk or be bedridden, so he will ask her why she is asking to go to the hospital when she is able to walk around. So if you are from a family of farmers or an illiterate family, the only time you come to the hospital is when you are really sick and you cannot move or are bedridden.* (Patient 2, Post-screening, asymptomatic, Interview)

Women perceived their compatriots as facing high hurdles in justifying their need for care to their husbands. However, when women talked about their own husbands, most reported that they had been supportive of them receiving care.

#### Sub-theme: cost for receiving treatment in Addis Ababa

While cervical cancer screening and treatment services are free in Ethiopia for insured and low-income women (upon proof of status), women described the costs of laboratory tests, drugs, as well as of transport to and housing in the capital, as a significant barrier to care. Two-thirds of women receiving care mentioned finances as a barrier, citing lack of money as a reason for delaying treatment and for being forced to undergo substandard care options.

*My big problem was financial problems—*‘My pocket was empty’*—I did not have cattle and farms for harvesting. I earned money from selling different drinks like coffee, tea and* ‘tella’ *but after I got sick and becomes bedridden, I did not have any source of income. My younger child suffered with me. He borrowed money from different people to treat me* (Patient 13, Treatment completed, Interview).

*The medication that I had prior to radiation was so expensive. One dose cost a 1550 birr and it could only be bought from abroad. So I was willing to pay for one or two doses, but they told me it had to be six consecutive doses and it could not be interrupted. So I told them I could not afford it and they changed my treatment to radiation.* (Patient 1, Currently in treatment, Interview)

Unless women had a provider or relative advocating for them and connecting them to social support services, they often went without care, as was the case for this patient:

*They told me to go to Addis Ababa and told me they were not able to help me there [at the facility]. And I have no money or energy to go to Addis Ababa… I just want to feel better and be healed here because I cannot go to where they referred me to and I wanted them to at least take pity on me and help me here.* (Patient 9, Awaiting treatment, Interview)

Women expressed a strong preference for receiving care close to where they live, especially given their compromised health. This patient’s statement reveals her fatigue and desire for emotional support from providers.

### Theme: sources of delay in accessing appropriate treatment once care is initiated

Patients and providers identified multiple sources of delay for patients in receiving care once they had initiated care.

#### Sub-theme: misdiagnosis and improper treatment

A notable source of delay was that many women were misdiagnosed during their initial assessments and had to make repeated visits, often to private facilities, before being correctly diagnosed.

*I …had stabbing pain on my waist and my lower abdomen. I was busy with work…so I just went to the pharmacy … he told me that it was nothing serious and he sold me Amoxicillin. I tell you it is these pharmacies that kill people.* (Patient 6, Treatment Completed, Focus Group)

Approximately 40% of women reported receiving an incorrect diagnosis or treatment. Some women reported being given medication such as antibiotics or analgesics during their initial visits rather than a referral*.*

*My doctor was also telling me I have a kidney infection and it was the case manager who was a friend who told me to go to Woliso hospital. So there was a lot of delay.* (Patient 7, Awaiting treatment, Focus Group)

Provider mistakes coupled with community stigma led women to feel alone in managing their illness.

*Mostly the problem is with the physicians in our area, they immediately give us medication but they do not tell us to go see a doctor as problems like cervical cancer could exist. If they had given us advice and told us what problems we had, we could have taken early action to protect ourselves... It is because of our own strength that we survive. Even the society stigmatizes you. But me personally I have convinced myself that I am ok and I will be ok, that is why I am alive today.* (Patient 7, Awaiting treatment, Focus Group)

It is not clear whether these mistakes were due to shortcomings in providers’ diagnostic skills or to providers’ desire to somehow help their patients despite the lack of appropriate treatment options at their facility. A few providers did note difficulties in diagnosing cervical cancer. They described challenges in distinguishing cervical cancer from sexually transmitted infections and noted the challenges in making accurate diagnosis without better training and a steadier flow of patients.

*When patients come with vaginal discharge, it would often be taken as sexually transmitted disease* (Physician 1, Health Center)

*Clients are usually misdiagnosed even with vaginal discharge; they would go back home after getting managed as an STI case.* (Health Officer 6, Health Center)

*We diagnose what we know. I may diagnose another disease as I don’t have the knowledge of cervical cancer…There is a knowledge and training gap. No exposure exists and we may misdiagnose cases. There is a fistula-trained expert here. When we are in doubt, we just consult and confirm [with him] for fistula cases. We don’t have the supplies and diagnostics for cervical cancer. We are not practicing screening. We need the training and to have a dedicated expert for that*. (Midwife 4, Rural Health Center)

#### Sub-theme: lack of equipment and resources

Most service delays reported by women receiving care occurred post-screening, during treatment. The most frequent reasons for delay were broken treatment equipment and a shortage of hospital beds. Providers rarely noted the lack of treatment and laboratory resources post-screening as a source of delay.

*Yes, it has been 15 days since the chemotherapy machine has been out of order and we cannot get treatment until it is fixed.* (Patient 6, Treatment completed, Focus Group)

*So that is where they took samples from and then …they sent [the sample] claiming that the equipment is not available here, so I had to wait for 2 months until I received the results. During that time my illness was getting worse and worse and then I had to wait another 3 months to get any kind of treatment after the diagnosis was made…so I waited for 5 months without any treatment.* (Patient 1, Currently in treatment, Interview)

#### Sub-theme: long wait times between appointments

A frequently mentioned source of delay in receiving care was the long wait times for follow up appointments. Providers noted that patients often were not able to attend referral appointments, often due to long wait times at crowded referral hospitals. This inability to make appointments was seen as contributing to delayed care and disease progression, particularly for poorer, rural women.

*Referred patients complain that they don’t get care on time. They may have long appointment waits while having advanced disease. I know some referred patients who died without getting the care due to long [waits for] appointments at the referral hospital.* (Health Officer 3, Rural Health Center)

Women receiving care also frequently noted the long wait times when asked directly about delays and noted the long times between appointments when describing their care history.

*When I am coming to get these treatments, I am given many [appointments] and long [waits between] appointments, and even worse you might not get any service when you come on the date of your appointment … Perhaps what I would have like to be done differently relates to … the lengthy appointments. I actually felt sad and emotional whenever they gave me appointments because I thought the time was too long.* (Patient 3, Treatment completed, Interview)

### Theme: patient-provider communication

#### Sub-theme: lack of communication

Not only was quality of care compromised by delayed and inaccurate diagnoses, but women receiving care also emphasized poor provider–patient communication as a source of delay and inadequate care. Although several patients had positive, supportive relationships with providers, most patients described either a lack of communication or poor-quality provider communications around their diagnosis and treatment. Some patients were never directly told of their diagnosis.

*R1: The doctor told me that I had to come to Addis Ababa to get the treatment and I knew immediately that what I had was cancer*.

*I: But did the doctor inform you clearly that it was cervical cancer*?

*R1: No, when I heard him saying that I had to have radiation treatment I concluded that I had cancer*.

*I: So the doctor did not say it was cancer*?

*R1: No he did not, and no one has told me still*. (Patient 7, Awaiting treatment, Focus Group)

#### Sub-theme: power imbalances

As indirectly shown above, both patients and providers mention that patients often do not feel comfortable asking questions. Patients revealed deep feelings of powerlessness and fear in the face of their diagnosis but also of provider authority. Some expressed fear of provider retaliation or provision of substandard care should they ask for more information or about alternatives to provider recommendations.

*I wanted to ask (the provider) if I am going to die. And I also wanted to ask if there is another alternative than the operation. … Yes, I am scared to ask questions because if I say something wrong, I am scared he will maybe cut me in the wrong place and not do his best.* (Patient 12, Awaiting treatment, Focus Group)

#### Sub-theme: language barriers

Language barriers also posed a problem for patients communicating with providers among the Oromo women interviewed, and they noted the importance of translators.

*At Black Lion Hospital I felt uncomfortable due to the language barrier. We [my providers and I] did not understand each other. Due to this reason there was a 2-month delay for my follow up and therapy.* (Patient 11, Awaiting treatment, Interview)

#### Sub-theme: patient shyness

While many providers acknowledged the difficulty that patients have in asking questions on topics where discussion is socially discouraged, some still placed responsibility on patients to raise issues and direct their own care.

*Maybe the difficulty lies with clients who fail to openly discuss health issues around such private body parts.* (Midwife 5, District Hospital)

### Theme: recommendations for improving care

#### Sub-theme: expand and decentralize screening, diagnosis, and treatment services

One of the most frequent suggestions by both providers and patients for improving care was to expand screening, diagnosis, and treatment services. Suggestions included having more trained providers and more treatment sites. Relatedly, many patients urged that more comprehensive services, especially diagnostic and preventative treatment services, be located closer to where patients live, rather than at referral hospitals. Providers also expressed frustration that that equipment and training did not reach the lower level facilities and that local and regional health officials did not appear to prioritize cervical cancer.

*There is a lack of human resource in the cancer field, you go to the hospital and you see the lobby full of people and only a few doctors to help them. And then these few specialists are always called in to meetings while the sick are waiting and then they are replaced by doctors who are not specialists to look after us and they just call us in and give us another appointment…And also if we could have cancer treatment centers in our regions so that we do not have to come all the way to Addis Ababa, that would be something. …* (Patient 14, Treatment completed, Focus Group)

*The [local] intervention sites need to be comprehensive with screening, lab confirmation, and treatment [services*]. (Midwife 6, District Hospital)

#### Sub-theme: increase community awareness of cervical cancer

The second most frequent suggestion for improving care, from both providers and patients, was to increase community awareness of cervical cancer and prevention through community health education and outreach.

*So I think the community needs to know that cervical cancer is not a punishment from God or an illness of sinners, so creating awareness about the illness is very important to change this wrong belief. … You see the government has not given cervical cancer a lot of focus because it is a non-communicable disease****.**** … It should be given coverage on all media just like other communicable illnesses as well.* (Patient 7, Awaiting treatment, Focus Group).

Providers also strongly felt that community education and awareness-raising would diminish stigma and make it easier for patients to talk with them about symptoms and concerns:

*“If awareness is created, patients would tell you their secrets, they (would) have trust in providers* (Midwife 7, District Hospital)

Patients noted that in order to decrease stigma, health education should include positive (as opposed to fear-based) messages that cervical cancer was preventable and treatable.

*It is very important that women are aware that the treatment exists and that they should get screened for cervical cancer now and then before they are really ill and it is too late* (Patient 3, Treatment completed, Interview).

#### Sub-theme: increase leadership on issue

Both patients and providers noted that cervical cancer health education is needed not only for women, but also health care providers and government leaders to increase a sense of urgency around the problem. Providers especially felt that cervical cancer was currently treated as a second-tier concern.

*To improve the quality, the region has to support the [cervical cancer] program well. There is no follow up; no one asks how the program is running, what has been done and how it is done* (Midwife 3, Referral Hospital)

*It [cervical cancer] needs the attention of decision makers. Not only at higher level, but also lower levels* (Health Officer 2, Urban Health Center)

*I know cervical cancer takes secondary status nationally* (Midwife 7, District Hospital)

### Triangulation of patient and provider themes

In general, the themes we found were common between providers and patients (see Table 5). The overlap was greatest in the recommendations for improving care. An interesting difference was that providers saw a lack of patient education as the primary barrier to care while patients emphasize their financial difficulties, perhaps reflecting the screening focus of providers, and the treatment focus of the women receiving care. In addition, while providers frequently noted poor follow-up and referral systems, patients did not mention problems with the components of these systems, only the wait times for receiving appointments. Although providers were aware that wait times were long for follow up and referral services, few mentioned the shortages of laboratory services or supplies, beds, and functioning equipment as the reasons for delays as patients did. Perhaps the most striking difference is on the issue of stigma, which patients repeatedly state as their primary challenge in living with cervical cancer and an important barrier for initiating and follow up care. Only one provider mentioned stigma as a patient challenge, and then, only in passing. Finally, on the topic of patient-provider communication, while both patients and providers were aware that patients had difficulty talking about cancer and reproductive health, providers seemed largely unaware of any shortcomings on their own part in communicating clearly with patients. Only one provider mentioned that he had difficulty discussing cancer with patients.

## Discussion

This study set out to describe the availability of cervical cancer care in our project area, the barriers to cervical cancer care in our sample, the sources of delay in obtaining care, and women’s perceptions and experiences of their care. We conducted interviews and focus group discussions with patients and providers at various levels of the cervical cancer screening, diagnosis, and treatment chain.

The characteristics of our study sample were consistent with national characteristics. For example, the youth of providers (80% of respondents < 30 years of age) was is in keeping with the youth of health care providers nationally, resulting from the country’s rapid expansion of health provider training [33] and the fact that the majority of providers were male reflects Ethiopia’s unusually high proportions of male non-physician health providers, including midwives, nurses, and health officers. The majority of women receiving care in our sample were within the age group found nationally to have peak incidence of cervical cancer [19], but younger than found by Gizaw et al. [18] at Tikkur Ambessa between 2008–2012. They were more likely to be divorced than is the case nationally for women ages 18–49 (20% vs. 6%) [34].

We find that women usually seek care when they have symptoms of advanced disease and that lack of funds is a major barrier to care. This finding is somewhat expected, as non-use of formal health care services when ill is common in Ethiopia, with the most frequent reason for non-use (over 40% of those asked) being the shortage of money [35]. Although cancer screening, diagnostic, and treatment services are included in Ethiopia’s Essential Health Service Package [36], are covered by the country’s community-based health insurance and social health insurance schemes, and are free for uninsured women designated as “poor” by their local leaders [37], travel and some ancillary care (e.g., medications and laboratory services) may not be covered or may only be available at private facilities. Our findings suggest that for uninsured women who do not meet poverty thresholds for free care, paying for care out-of-pocket may be prohibitively expensive. Medical and other costs to patients for obtaining treatment for cervical cancer are high [38], and have been found elsewhere to reduce the ability to adhere to treatment [39]. As Ethiopia moves toward universal health coverage, we recommend that policymakers review public insurance schemes to ensure that they are aligned with the Essential Health Service Package and redouble efforts to increase enrollment in these plans that still only cover a small fraction of the country’s population. We also suggest that they examine ways to ensure that cervical cancer diagnostic, pharmaceutical, and social support services are available at a wider range of public facilities so that women are not forced into expensive private care. Expanding services may require new revenue-generating mechanisms such as tobacco, alcohol, and sugar-sweetened beverage taxes, which can also play a role in preventing non-communicable diseases [40].

### Gender implications

As women tend to access care when ill even less than men in Ethiopia, even though they experience greater disease incidence [35], the need to increase broad community awareness of the seriousness of cervical cancer and of the remedies is critical. Public awareness campaigns may, in part, help shift some men’s view that women should only access treatment when they are bedridden, as described by respondents. Family-focused campaigns that encourage young women who are receiving vaccinations to encourage their mothers to be screened may also be a promising strategy for increasing demand for screening services. The tone of such campaigns should be actively welcoming and anti-stigmatizing as women may be particularly prone to both perceiving and holding stigmatizing attitudes about cancer [41].

Relatedly, our findings here suggest that gender power relations affect women’s access to cervical cancer services in terms of limited female intra-couple decision-making power, social norms such as modesty that discourage use of services, and health worker attitudes and behavior; among others. Beyond health system shortcomings and high out-of-pocket costs, on the demand side, the combination of gender and age are also likely behind women’s delayed ability to access care. In particular, we note that older women may disproportionately face structural disadvantages, such as low levels of education, or an inability to speak the dominant language of the area, that limit their access to care and hamper their communication with providers [41].

The continuation of public campaigns such as Ethiopia’s Girl Effect-Yegna program [42] that promote dialogue about women’s rights and health, might encourage women to talk more openly about their health needs and experiences. Increasing the availability of more community-based screening and self-care strategies, for example HPV-DNA self-sampling, might also mitigate modesty concerns, negative provider-patient gender dynamics, and the need to negotiate care with partners. In designing these interventions, policy makers should be sensitive to the ways in which gender, age, class, and location can intersect to make certain women particularly vulnerable to poor care [43].

### Stigma

Patients highlight the delay in care seeking due to societal stigmatization of cervical cancer for its perceived linkages with (female) sexual promiscuity. Studies elsewhere in sub-Saharan Africa [41, 44] and in Ethiopia [16, 22, 45] have found women to hold stigmatizing beliefs about women who develop cervical cancer, and thus about screening and treatment. Expanded cervical cancer health education campaigns should be designed to reduce the existing levels of stigma around the disease and to correct the misconception that women in monogamous long-term relationships are not also at risk. In addition to health education, increased access to cervical cancer treatment services likely can have the allied benefit of improving women’s screening uptake by reducing the stigma surrounding the disease [46]; as has been the experience in sub-Saharan Africa with HIV/AIDS [47, 48].

### Service availability

Much remains to be done to adequately meet even the currently low levels of demand for services, however. Only a handful of the facilities sampled in this study were providing screening services, a finding in keeping with a recent Ministry of Health report that found that only 16% of health facilities in the country were offering screening [49]. Moreover, as other studies in low-income settings have found provider knowledge of cervical cancer screening and treatment best practice is patchy, particularly for mid-level providers [17, 24, 28, 30, 50–54]. For example, an internal evaluation in 2015 that we conducted at Debre Markos Referral Hospital (one of the facilities sampled in this study) found that knowledge of different screening and treatment procedures was inconsistent and varied significantly by profession with only 57% of respondents in able to identify cryotherapy as a treatment method, and only 48% of nurses able to identify VIA as a screening method, even though these are recommended practices in Ethiopia [29]. Almost 40% of nurses and midwives in that evaluation were not able to identify vaginal discharge as a symptom of cervical cancer; one-third could not identify abdominal pain, and almost 70% of all midlevel providers were not aware that cervical cancer could be asymptomatic [29]. Providers’ poor knowledge of symptoms and lack of knowledge of appropriate protocols may explain why so many of the women in our sample were initially misdiagnosed. Our findings and those of other studies suggest the need to intensify in-service training for providers, especially mid-level providers in primary level facilities, focusing initially on alerting them to the range of cervical cancer symptoms. Pharmacists should also receive training in cervical cancer symptoms as our findings suggest that they may be an initial point of contact for patients.

We find that screening, diagnosis, and treatment services are still scarce outside of large referral hospitals and that travel to these services is a major barrier to receiving care. It seems that low availability of diagnostic and treatment services–due to shortages of beds, broken equipment, and lack of laboratory supplies—are significant bottlenecks in the care continuum. Other studies have found similar gaps in treatment infrastructure in Ethiopia [17, 55]. These findings suggest both the validity of the current MOH strategy to decentralize care and treatment services to district-level facilities as well as the need to redouble efforts to implement it. Better distribution of screening and supplies to lower-level facilities, the designation or construction of spaces for private exams, and greater attention to maintenance of treatment equipment and supply of consumables are also a priority for increasing coverage.

### Patient-provider communication

Finally, while implementation of these recommendations may go some way to improving the working environment for providers and thereby their interaction with patients; our study points to the continued need for training around patient rights and respectful communication with patients. Many of our patients were either not told their diagnosis or did not have it clearly explained to them, and several women reported disrespectful encounters with providers, particularly during treatment. While women also told stories of extraordinary providers who went out of their way to follow their care, our findings suggest ongoing problems with patient-provider communication that often further delays care.

As noted in our results, the most striking thematic divergence between patients and providers was stigma, which was central to women’s care experience but rarely mentioned by providers. The lack of provider insight into the factors driving women’s care-seeking behavior and the patient-provider communication problems discussed above could be partially addressed by including patient narratives in cervical cancer refresher and in-service training. Such training could also have discussions of stigma and embarrassment as well as skills-building, counseling exercises for communicating with women on these topics. Training alone will be insufficient to improve the quality of care. The cumulative weight of health systems barriers (e.g., the absence of supplies and equipment) that providers face in delivering the care they know is appropriate, may discourage them from offering counselling and moral support. Therefore, promoting respectful, patient-centered care will also require policies to address these barriers.

### Limitations

This study has several limitations. It was designed as an exploratory pilot study to collect preliminary data on service availability for a larger study on multi-level cancer care in low-income countries. As such, it had a modest sample size for women seeking care, and a limited geographic scope, which limits its generalizability. However, our sampling aim was not to recruit a large enough sample to ensure representativeness but rather, as a qualitative study, to recruit a sample sufficient to reach data saturation. Both the theoretical literature and empirical studies suggest that the number of women we interviewed was sufficient for reaching saturation [56–60]. We feel confident that we reached data saturation with both the sample of 30 healthcare providers and the sample of women who had entered the care with advanced disease.

Nevertheless, we note that recruitment of recently screened or diagnosed women at health facilities in East Gojjam Zone was much slower than anticipated. Very few women presented for care during the study period at the primary care facilities where we did the bulk of our recruitment. The low numbers of women presenting could be the result of women seeking care rarely and only when severely ill as discussed above. It could also be that fear of stigma or beliefs about service availability led women to bypass local primary care facilities in favor of better resourced, and more anonymous referral hospitals. Even after extending recruitment several months, we were not able to recruit as many patients at the early stages of care as we would have liked (we reached only 50% of our target), and, as a result, we were not able to reach saturation in our analysis of women who had recently been screened or diagnosed. As noted above, very few women in Ethiopia are currently being screened, and most women seek care only when severely ill. Therefore, to fully capture the early screening and diagnosis experiences of patients in Ethiopia, particularly asymptomatic women, we recommend that researchers allocate a significant amount of time for recruitment and data collection and explore different study designs and recruitment methods for this currently small and hard-to-reach population. Although we could decern no difference in the themes that emerged from interviews with women in the early or late stages of care, it is possible that their care patterns differ. Women in the early stages of care could face fewer delays than women with more advanced disease because the care they require might be less specialized and more available than cancer treatment. However, it is also plausible that these women might experience even more severe delays and poor provider communication than symptomatic women because their cases are not seen as urgent. This question is an important area for future research.

Finally, we note that interviewer quality varied significantly in our study, which may have compromised the richness of the data. Both the relative skill of our focus group facilitator and the sharing encouraged by the format led to more detailed data emerging from the focus groups than interviews and overall, the quality of the interviews and focus groups with women were much richer and more detailed than our interviews with healthcare providers.

## Conclusion

This qualitative study assessed experiences in receiving and providing cervical cancer care among a sample of Ethiopian providers and patients. Our main findings were that patients experience bottlenecks and delays at each stage of care mostly due to health system weakness and poor patient-provider communication, and that providers face significant training and health system barriers that prevent them from offering care.

Low perceived risk of cervical cancer, high stigma, and a lack of knowledge about cervical cancer among both providers and patients were significant sources of delay in initiating and following up care. Few patients had been aware of cervical cancer pre-diagnosis. Those who were aware assumed that they were not at risk because they were monogamous or not sexually active. Providers report being poorly trained or equipped to screen and diagnose patients, and patients report misdiagnosis as a common source of delay. Once diagnosed, women faced multiple-month delays after referrals, and, once in treatment, broken equipment, lack of laboratory supplies, and a shortage of hospital beds resulted in additional delays. The most frequently mentioned barriers to cancer treatment were a lack of housing and travel funds while receiving care in the capital. By interviewing both patients and providers we uncovered important discrepancies in understanding of the barriers to care, appreciation of the importance of stigma in delaying care, and awareness of poor patient-provider communication in care.

Our findings suggest the need to intensify in-service training for mid-level providers, focusing initially on alerting them to cervical cancer symptoms, informing them on issues of stigma and embarrassment among women requiring care and strengthening counseling skills for sensitive topics. Better distribution of screening and diagnostic supplies to lower-level facilities and better maintenance of treatment equipment at tertiary facilities are also a priority.

## Data Availability

The datasets generated and analyzed during the current study are not publicly available due to restrictions of our Institutional Review Board, but are available from the corresponding author on reasonable request.

## Abbreviations

DMU: Debre Markos University
DMRH: Debre Markos Referral Hospital
HIV/AIDS: Human immunodeficiency virus/acquired immunodeficiency syndrome
HPV: Human papilloma virus
MWECS: Mathiwos Wondu-YeEthiopia Cancer Society
VIA: Visual inspection of the cervix with acetic acid
